# In Vitro Photodynamic Effect of Phycocyanin against Breast Cancer Cells

**DOI:** 10.3390/molecules21111470

**Published:** 2016-11-03

**Authors:** Subramaniyan Bharathiraja, Hansu Seo, Panchanathan Manivasagan, Madhappan Santha Moorthy, Suhyun Park, Jungwan Oh

**Affiliations:** 1 Marine-Integrated Bionics Research Center, Pukyong National University, Busan 608-737, Korea; sbrbtc@gmail.com (S.B.); manimaribtech@gmail.com (P.M.); santham83@gmail.com (M.S.M.); 2 Department of Biomedical Engineering and Center for Marine-Integrated Biotechnology (BK21 Plus), Pukyong National University, Busan 608-737, Korea; biomed84@hanmail.net; 3 Department of Biomedical Engineering, University of Texas at Austin, Austin, TX 78712, USA; tumbler77@gmail.com

**Keywords:** phycocyanin, photodynamic therapy, laser therapy, reactive oxygen species

## Abstract

C-phycocyanin, a natural blue-colored pigment-protein complex was explored as a novel photosensitizer for use in low-level laser therapy under 625-nm laser illumination. C-phycocyanin produced singlet oxygen radicals and the level of reactive oxygen species (ROS) were raised in extended time of treatment. It did not exhibit any visible toxic effect in the absence of light. Under 625-nm laser irradiation, c-phycocyanin generated cytotoxic stress through ROS induction, which killed MDA-MB-231 breast cancer cells depending on concentrations. Different fluorescent staining of laser-treated cells explored apoptotic cell death characteristics like the shrinking of cells, cytoplasmic condensation, nuclei cleavage, and the formation of apoptotic bodies. In conclusion, phycocyanin is a non-toxic fluorescent pigment that can be used in low-level light therapy.

## 1. Introduction

Photodynamic therapy (PDT) is emerging as a non-invasive novel therapy to treat cancer, psoriasis [1], and other kinds of infections. It has many advantages over conventional chemotherapy and surgical resection, such as localized eradication, not affecting the connective tissue in the treatment area, and overcoming drug resistance mechanisms [2]. The PDT process is performed with the aid of light and photosensitizers and further photochemical reaction leads to cell death [3]. Upon light exposure, the photosensitizers excite from their ground state to a higher state and subsequently transfer the absorbed photon energy to the adjacent molecules to generate cytotoxic potent free radicals. The photosensitizers can initiate free radical generation through two main pathways: (i) transferring the excited energy directly to surrounding oxygen molecules to generate highly reactive singlet oxygen radicals (^1^O_2_), which is termed as a type II mechanism; or (ii) the excited energy is transmitted to other molecules to propagate hydroxyl radials (OH), hydrogen peroxide (H_2_O_2_), and all other kinds of radicals, including oxy radicals, which is deemed as type I mechanism [4]. The systematically-generated free radicals stimulate cytotoxic stress, which leads to cell cycle arrest, apoptosis, or necrosis [5]. Many of the currently practicing photosensitizers like protoporphyrin IX and chlorin e6 are hydrophobic and they easily aggregate in physiological solution which, leads to a reduction of PDT efficiency [2]. Natural water-soluble pigments are needed due to their biological effects, biocompatibility, and easy clearance from the biological system.

Phycocyanin is one of the natural biological molecules found in *Spirulina platensis* [6] as a light-harvesting pigment, which possess anti-inflammatory, anti-cancer, and anti-oxidant effects [7]. It is an intense blue-colored fluorescent molecule [8], which shows good photostability, and it has been widely used in food and cosmetic products [9]. Phycocyanin has many advantages, including water solubility, a non-toxic nature [10], and immune system boosting properties [11]. Zheng et al. stated that normal cells can easily metabolize and clear the phycocyanin sooner than cancerous cells [12], which may help to eradicate cancer cells selectively through PDT. Even though phycocyanin is recognized as a photosensitizer, it has not been utilized well as a PDT drug compared to porphyrin-based pigments. Phycocyanin belongs to the biliprotein family, based on its light absorption property it has been classified into three types, such as (i) c-phycocyanin; (ii) c-allophycocyanin; and (iii) c-phycoerythrin. In the present study, we have chosen c-phycocyanin for its various biological effects and photostability.

Low-level laser therapy (LLLT) is an emerging technology to treat different conditions, such as myocardial infraction, traumatic brain disorders, and tumor eradication [13]. The LLLT system has been referred to as a non-invasive and non-thermal irradiation that does not cause much pain or inflammation to the treated tissues. LLLT has been used in clinics to treat various kinds of skin diseases, including acne, herpes virus lesions, and hypertrophic scars [13]. The usage of LLLT has been increased compared to other laser therapies that use high-power energy to destroy the tumor through its heating effect. In the present investigation, we studied the effect of c-phycocyanin-mediated photodynamic effect on breast cancer cells using a 625-nm LLLT system.

## 2. Results and Discussion

**Scheme 1:**
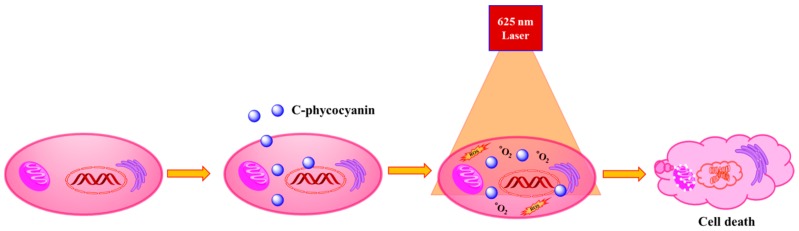
Systematic representation of c-phycocyanin uptake by MBA-MD-231 cells and subsequent photodynamic therapy using a 625-nm laser.

**Figure 1:**
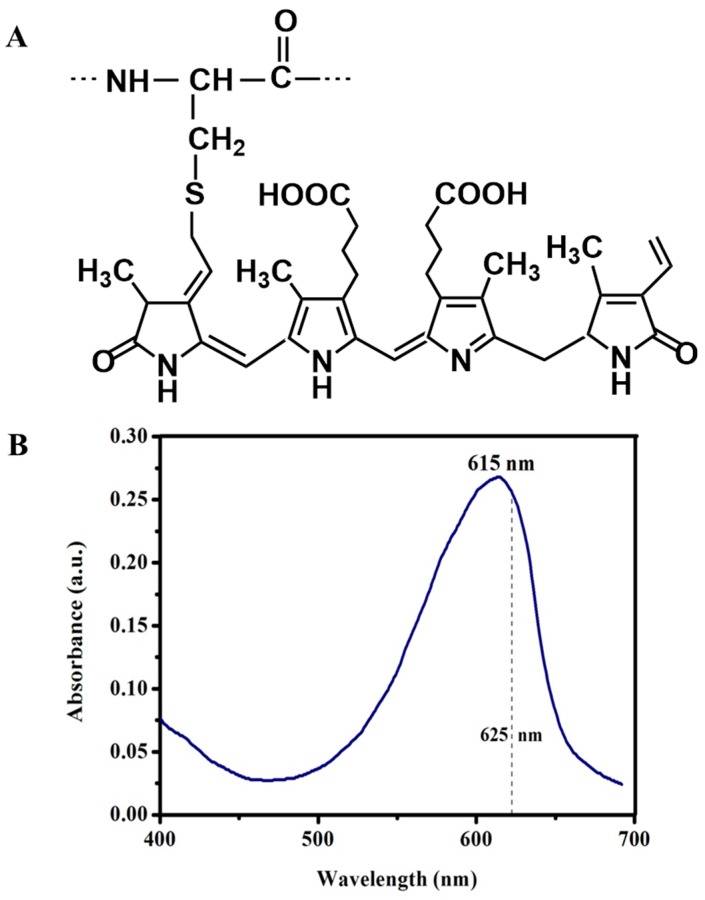
Chemical structure and absorbance spectrum; (**A**) Chemical structure of c-phycocyanin; and (**B**) UV-VIS absorbance spectrum of c-phycocyanin dissolved in water at the concentration of 10 μg·mL^−1^.

**Figure 2:**
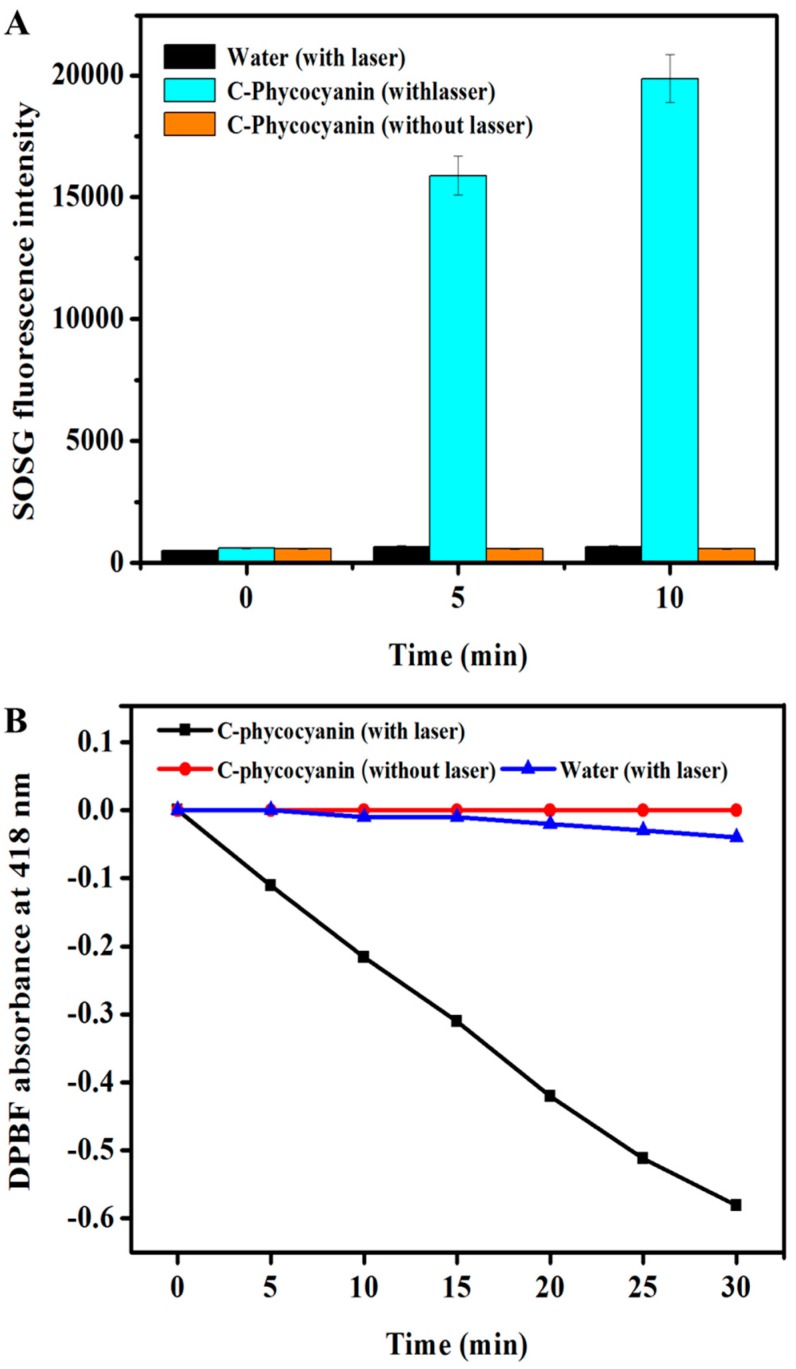
Screening of free radical generation efficacy by c-phycocyanin under 625-nm laser illumination at 80 mW·cm^−2^ using SOSG (**A**) and DPBF (**B**) assays.

**Figure 3:**
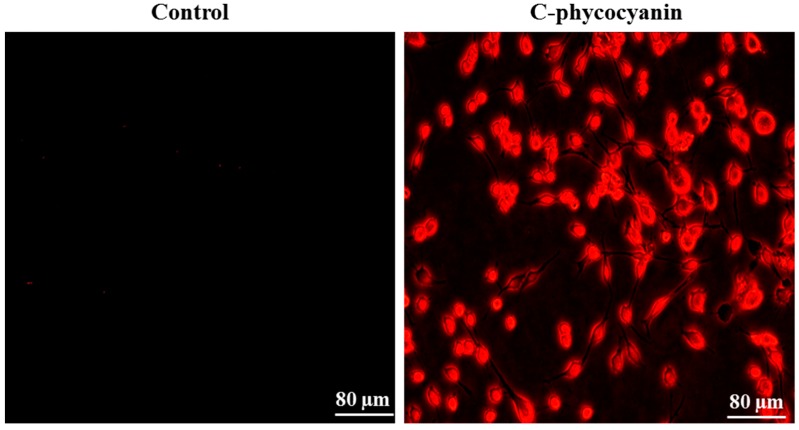
Fluorescence microscopic images demonstrate cellular uptake of c-phycocyanin by MBA-MD-231 using an excitation/emission range of 600/650-nm.

**Figure 4:**
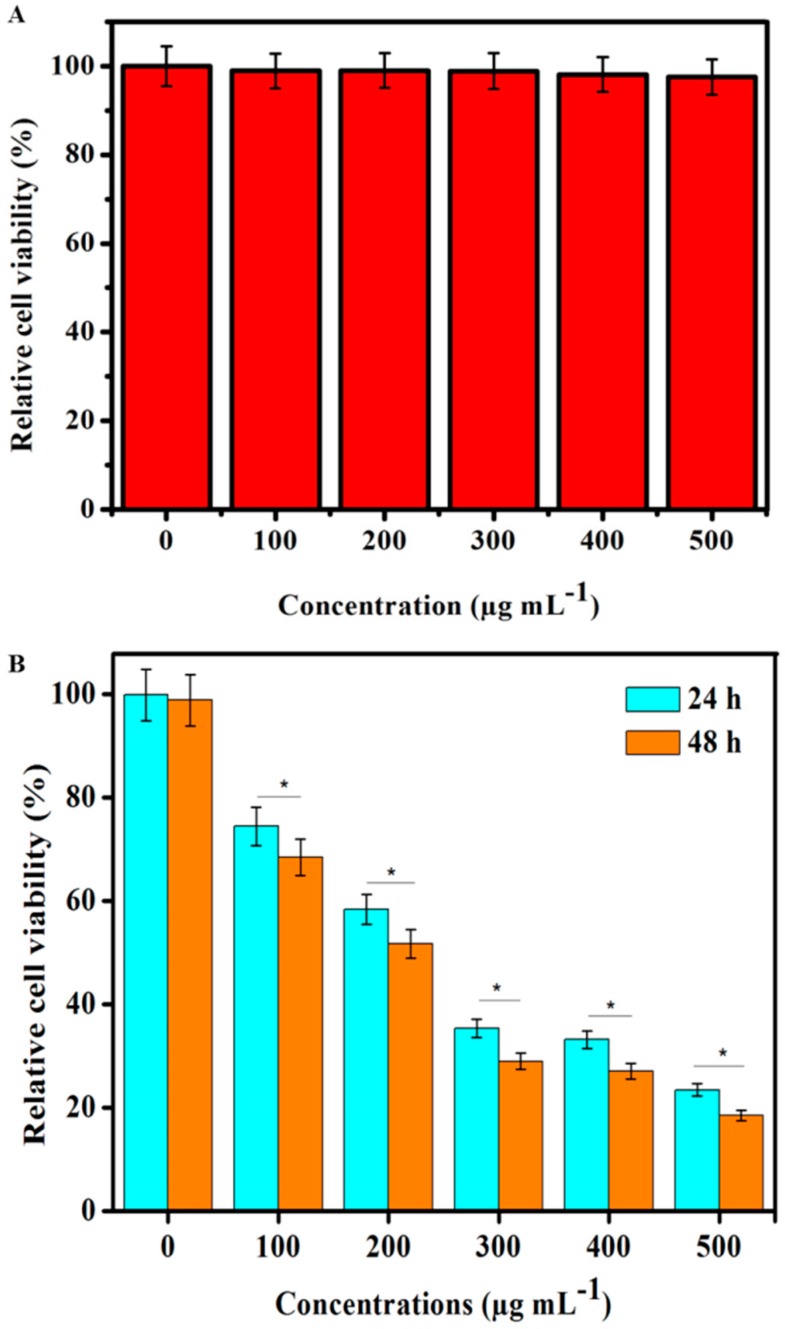
MBA-MD-231 cell viability (**A**) Biocompatibility of c-phycocyanin with MBA-MD-231 cells; and (**B**) different concentrations of c-phycocyanin-treated MBA-MD-231 cells showed cytotoxicity after PDT treatment using a 625-nm laser at 80 mW·cm^−2^ for 30 min. Data is expressed as mean ± SD of the three experiments. Percentage of cytotoxicity is expressed relative to untreated controls (* significant *p* < 0.05).

C-phycocyanin is a biliprotein seen in blue-green algae, which possess potential biological activities. Even though c-phycocyanin has been documented as a photosensitizer in a few studies, it has not been utilized much when compared to chlorin e6, a derivative of chlorophyll pigment. Scheme 1 explains the possible cellular uptake of phycocyanin by MBA-MD-231 cells and PDT treatment-induced cell death through the generation of ROS. C-phycocyanin is a major water-soluble pigment and the chemical structure of c-phycocyanin is shown in Figure 1A. It shows an absorption peak around 615-nm (Figure 1B). The 625-nm absorption intensity was marked and the same level laser system was used for the PDT experiment. C-phycocyanin is a novel class of fluorescent dye, which propagates singlet oxygen radicals upon 625-nm laser illumination, as evidenced from singlet oxygen sensor green (SOSG) assay (Figure 2A), the increasing level of SOSG green intensity indicates the increasing level of oxygen radicals. The SOSG fluorescent level was not raised in water samples or in laser-untreated c-phycocyanin samples, which confirms that singlet oxygen was generated only under laser exposure. The result of SOSG was in coherence with 1,3-diphenylisobenzofuran (DPBF) assay, where c-phycocyanin-mediated ROS generation was proven by the significant bleaching of DPBF absorbance at 418 nm (Figure 2B). DPBF assay was performed to confirm the result of SOSG on singlet oxygen generation. C-phycocyanin was effectively taken up by MBA-MD-231 cells within 6 h (Figure 3). Cellular uptake of c-phycocyanin was screened based on the excitation and emission range of c-phycocyanin around 600/650-nm. The photograph of control cells did not show any red signal and treated samples showed red colored cells, which proved the uptake of c-phycocyanin by MBA-MD-231 (Figure 3). As shown in Figure 4A, phycocyanin did not exhibit any visible cytotoxic effect on breast cancer and HEK-293 cells (Figure S1), even at a concentration of 500 μg·mL^−1^. C-phycocyanin has already been reported for its hepatoprotective effects [14] and it is safe and non-toxic to human health [15]. Generally, excited singlet oxygen will stimulate oxidative stress and affect cellular macromolecules to induce apoptosis or necrosis [16]. The cellular defense mechanism detoxifies these radical species, but the redox balance could be disturbed by overproduction of free radicals by continuous irradiation under the LLLT therapy system. The result of cell viability assay (Figure 4B) was in coherence with SOSG and DPBF assays (Figure 2). The PDT treatment caused cell death in both cell models through c-phycocyanin-mediated ROS production upon laser treatment (Figure 4B and Figure S1). Almost 62% of cell death occurred at 300 μg·mL^−1^ in MBA-MD-231 cells. The rate of cell death differed in HEK-293 cells where only 35% of cell death occurred at the concentration of 300 μg·mL^−1^. The cellular uptake efficiency of c-phycocyanin and its metabolism may vary between different cells [17]. Thus, the rate of cell death differed between MBA-MD-231 and HEK-293 cells. Ohyashiki et al. [18] demonstrated that DPBF fluorescence decreased in increasing concentrations of singlet oxygen radicals. Riesenberg et al. reported that a clinically-practicing drug, 5-aminolevulinic acid (5-ALA), induced photodynamic cell death at the concentration of 100 μg·mL^−1^ using a 635-nm laser. However, at the same time they observed reduced mitochondrial activity when the dose of 5-ALA impeded 2.5 mg·mL^−1^ without light illumination [19]. The use of higher concentration of c-phycocyanin (200 mg·Kg^−1^) has been reported as safe and it cures cerebral ischemia [20]. The formation of singlet oxygen depends on the concentration of the photosensitizer, the intensity of the laser light, and the amount of oxygen present in the treated environment. The temperature level was not altered until the end of a 30 min under the 625-nm LLLT system (Figure S2). We operated the LLLT system with a 1 min interval for every 5 min of treatment to avoid the heating effect.

### 2.1. Cellular Morphology

**Figure 5:**
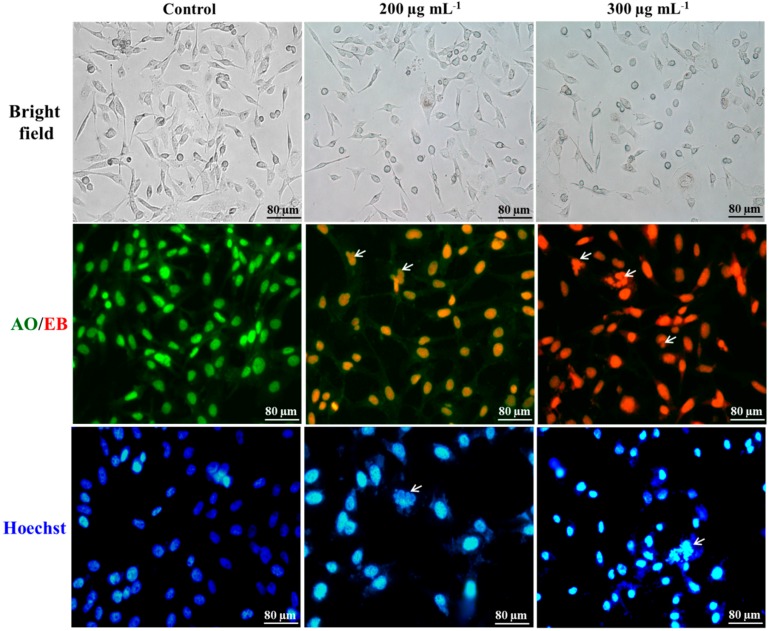
Bright field and fluorescent microscopic images of PDT-treated MBA-MD-231 cells with or without phycocyanin using 625-nm laser irradiation for 30 min at a power density of 80 mW·cm^−2^.

The c-phycocyanin-mediated LLLT-treated cells were examined under light microscope to observe treatment-induced alteration in cellular morphology. Photographs of control cells showed normal and uniform shape (Figure 5). Based on light microscopy studies, the disturbed morphology of MBA-MD-231 cells was confirmed in c-phycocyanin photosensitized cells under laser treatment. The cytoplasm was condensed and many of the cells were shrunk by c-phycocyanin sensitized laser treatment. Further different cellular organ specific cell staining was performed to analyze the treatment effect on breast cancer cells.

### 2.2. Differentiation of Cell Death

Acridine orange-ethidium bromide (AO/EB) staining was carried out to distinguish the cell death. Control cells appeared in green fluorescence with uniform morphology without any alteration. AO is a vital dye that stains both live and dead cells. However, EB is permeable only into the cell, which has lost its membrane integrity. The cell structure shrunk and is seen in a yellow-orange and red color, which indicates cell death in the treatment group (Figure 5). Intense red colored cells appeared highly in 300 μg·mL^−1^ c-phycocyanin-treated cells (Figure 5) due to the high accumulation of EB stain. The yellowish-orange colored cells (Figure 5) appeared in 200 μg·mL^−1^ treatment when green colored cells accumulated with EB. Clear, fragmented nuclei and cells appeared in the c-phycocyanin laser-treated group (marked by the arrow), which is a characteristic of apoptosis, a programmed cell death process.

### 2.3. Nuclei Cleavage

Hoechst 33258 staining revealed a major morphological change in the nuclei of PDT-treated cells. Hoechst is a cell-permeable stain that binds with the minor groove region of DNA and gives intense blue color emissions based on their accumulation. Nuclei of control cells were normal without any alterations (Figure 5), but the nuclei of c-phycocyanin-sensitized laser-treated cells were disturbed. C-phycocyanin-sensitized cells could generate ROS under laser exposure, which could attack the membraned organs inside the cells. Photographs of Hoechst stains clearly display the broken nuclei represented by arrows (Figure 5), which is a hallmark event of apoptosis. During the apoptotic process, an irreversible chromosomal condensation takes place, leading to the fragmentation of nuclei, a process called as karyorrhexis [21].

### 2.4. Intra-Cellular ROS Generation

**Figure 6:**
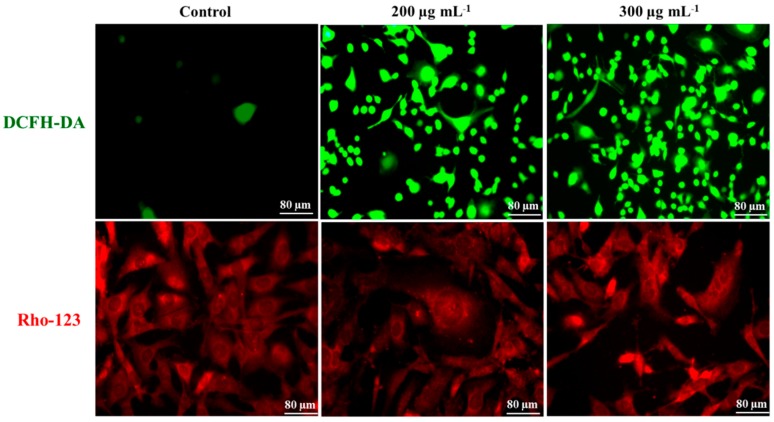
DCFH-DA staining of MBA-MD-231 cells following 1 h of PDT treatment with or without phycocyanin. Rho-123 staining demonstrated loss of mitochondrial membrane potential after 24 h of PDT treatment. The PDT treatment was performed using 625-nm laser for 30 min at 80 mW·cm^−2^ power density.

The PDT-generated intracellular ROS was measured using 2′-7′-dichloro dihydro fluorescein (DCFH-DA) staining after 1 h of laser treatment. The bar graph in Figure S3 represents the increased fluorescence level of dichlorodihydrofluorescein (DCF), which means that the ROS level increased depending on the concentration of c-phycocyanin under laser exposure. Compared to control cells, c-phycocyanin-treated cells emitted a greener signal, which indicated ROS generation upon 625-nm laser irradiation. The cell images (Figure 6) consistently supported fluorescent measurement in the microplate reader. The control cells did not exhibit much green fluorescence when compared with c-phycocyanin-treated cells. The results of DCFH-DA supported the output of SOSG and DPBF assay. DCFH reacts with all kind of intracellular ROS whereas SOSG only can react with singlet oxygen radicals and emits green fluorescence. ROS generation can interrupt the regulated metabolic process inside the cells and affect the cell proliferation cycle [22]. This was predicted as c-phycocyanin induced both type I and type II photochemical reactions.

### 2.5. Mitochondrial Membrane Potential

Figure 6 showed the depolarized mitochondrial membrane structure in c-phycocyanin-photosensitized MBA-MD-231 cells. The quantitative analysis of Rho-123 fluorescence intensity was shown in Figure S4. It represents that the mitochondrial membrane sequestration of rhodamine-123 (Rho-123) has been decreased in c-phycocyanin-sensitized PDT treated cells. The structure of mitochondria was normal and seen uniformly in control cells (Figure 6). Rho-123 is a cationic lipophilic dye readily sequestered by the mitochondrial membrane and reveals structural information under fluorescence microscopy. The disturbed mitochondrial membranes release pro-apoptotic factors, like BAX and BAK, which leads to apoptotic cell death [23]. The disturbed mitochondria promote the intrinsic mediated apoptosis through activation of caspase-8, which is present in cytoplasm [24]. The ROS propagation inside the cells triggers mitochondrial membrane permeabilization and enhances the ROS level further. Since mitochondria is involved in the electron transport chain [25], its breakage further enhances the ROS level in PDT treatment. Generally, ROS occurs as a byproduct of oxidative metabolism in mitochondria [26] and when the laser treatment generates ROS, the normal physiological redox system fails and leads to mitochondrial rupture [27]. Significant loss of mitochondrial potential further increases the ROS and releases cytochrome C from the mitochondrial intermembrane space, which activates the caspase-dependent apoptosis pathway [28]. Phycocyanin-related biliproteins are used to sensitize metal nanoparticles for different applications, such as anticancer and antimicrobial treatment [29]. Thus c-phycocyanin pigment can be extended to further research with nanomaterials for different kinds of biomedical applications.

### 2.6. Apoptosis Assay

**Figure 7:**
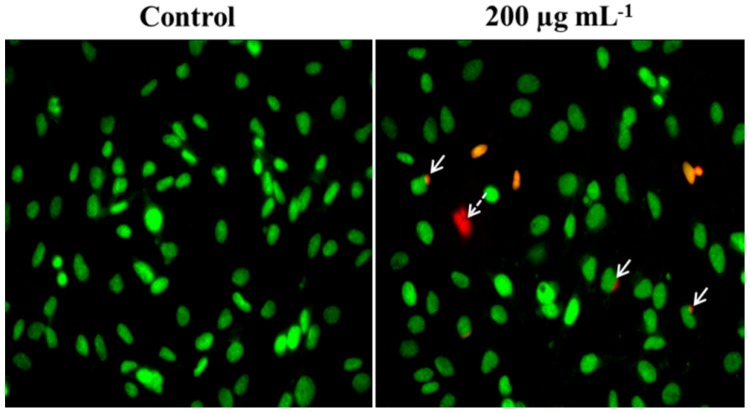
Annexin V-Cy3/6-CFDA staining of MBA-MD-231cells after 6 h of PDT treatment with or without phycocyanin, using a 625-nm laser at a power density of 80 mW·cm^−2^ for 30 min.

Annexins are a group of proteins that bind to phospholipids and can differentiate apoptotic cell death. The results of Annexin V-Cy3 revealed that PDT therapy induced apoptotic cell death. The transformation of phosphatidylserine from the inner membrane to the outer surface of the cells was detected through the binding of Annexin V-Cy3, which appeared as a yellow color (Figure 7). In addition, formation of apoptotic bodies were observed. The phosphatidylserine translocation was indicated by arrows and apoptotic bodies were pointed by tailed arrows in Figure 7. The control cells appeared in a uniform shape with 6-carboxyfluorescein diacetate (6-CFDA) stained green in color. C-phycocyanin-induced apoptosis was reported by Kong et al. in HeLa cells through ROS generation [30]. The mitochondrial permeabilization by ROS leads to cell membrane translocation and causes caspase-dependant apoptosis [31]. The Annexin-V staining revealed the phosphatidylserine externalization, a hallmark event of apoptosis.

## 3. Methodology

### 3.1. Materials

C-phycocyanin, 3-(4,5-dimethylthiazol-2-yl)-2,5-diphenyltetrazolium bromide (MTT), and staining components, such as acridine orange, ethidium bromide, Hoechst 33342, rhodamine-123 (Rho-123), 2′,7′-dichloro-dihydro-fluorescein diacetate (DCFH-DA), 1,3-diphenylisobenzofuran (DPBF), and Annexin V-Cy3 apoptosis detection kit were purchased from Sigma-Aldrich (St. Louis, MO, USA). Singlet oxygen sensor green (SOSG) was obtained from Thermofisher Scientific (Waltham, MA, USA). All of the chemicals purchased were used as received without further purification.

### 3.2. SOSG Assay

Two different methods were followed to confirm singlet oxygen generation using SOSG and DPBF assay. In the SOSG experiment, the measurement was based on green fluorescence emission of the dye upon its reaction with singlet oxygen radicals. To estimate the SOSG assay, approximately 10 μg·mL^−1^ of c-phycocyanin was used as experimental control concentration, to which 2.5 μM of freshly prepared SOSG was added in a 96-well microplate. In addition, water without phycocyanin was also taken for comparative analysis. The total volume of each well was adjusted to 200 μL using distilled water. The sample containing plate was irradiated under a 625-nm LLLT system at 80 mW·cm^−2^ power density for 10 min, with a 1 min interval for each 5 min illumination. The SOSG fluorescence intensity was measured using a microplate reader (Tecan Infinite F200, Mannedorf, Switzerland) every 5 min.

### 3.3. DPBF Assay

The generation of ROS efficacy by c-phycocyanin under 625-nm laser exposure was cross-checked using DPBF assay according to a previous protocol [32]. The fluorescence of DPBF decreases due to its reaction with singlet oxygen. C-phycocyanin at the concentration of 10 μg·mL^−1^ was put in a cuvette to which 10 μM of DPBF dissolved in DMSO was added. After measuring the initial absorption without a laser, the samples were illuminated under a 625-nm LLLT system for 30 min. A one minute gap interval was given for each 5 min of laser exposure. The sample not irradiated by the laser was kept as a control. The absorption of the control and treated samples were recorded at 418 nm for 5 min each.

### 3.4. Cellular Uptake

To find the cellular uptake activity of c-phycocyanin by MDA-MB-231 cells, cells were seeded in a 12-well plate at a density of 1 × 10^5^/well. The seeded cells were treated with 10 μg·mL^−1^ of c-phycocyanin separately. After 6 h of incubation, the cells were washed with phosphate-buffered saline (PBS) to remove the free c-phycocyanin molecules, and the cells were mounted under a fluorescence microscope using an excitation/emission range of 600/650-nm.

### 3.5. In Vitro Toxicity

Two different cell line models were used in the present study. The first is MBA-MD-231 breast cancer cell line, and the other is normal human embryonic kidney-293 cells (HEK-293). The pre-seeded cells at a concentration of 1 × 10^4^ cells/well were treated with different concentrations of c-phycocyanin for 24 h. Treatment of each concentration was repeated three times. Cells incubated without phycocyanin were maintained as a control. The media was then removed and cells were incubated with MTT stain (0.5 mg mL^−1^) for 2–4 h, and the medium containing the MTT stain was removed. Finally, a crystal formazan purple-colored product formed inside the living cells was dissolved with dimethyl sulfoxide (DMSO), and the color intensity was measured at 570-nm to calculate the cell viability using following formula:

(1) $$\%\;\mathrm{of}\;\mathrm{cell}\;\mathrm{viability}\;=\;\frac{\mathrm{OD}\;\mathrm{value}\;\mathrm{of}\;\mathrm{treated}\;\mathrm{samples}}{\mathrm{OD}\;\mathrm{value}\;\mathrm{of}\;\mathrm{control}\;\mathrm{samples}}\;\times \;100$$

### 3.6. In Vitro PDT Cell Toxicity

MDA-MB-231 cells and HEK-293 cells were seeded in 96-well plates separately at the concentration of 1 × 10^4^ cells/well and treated with different concentrations of c-phycocyanin ranging from 100–500 μg·mL^−1^ for 6 h. The free c-phycocyanin molecules were then removed by washing the cells with PBS. The cells with 100 μL of PBS were kept at 37 °C in a CO_2_ incubator for 30 min. Then cells were illuminated under 625-nm laser light with 80 mW·cm^−2^ power density for 30 min. One min intervals were set for every 5 min of treatment to avoid the heating effect. After laser treatment, the cells were again incubated for another 24 h, and MTT assay was performed to determine the PDT-mediated toxicity effect on treated cells. Cells exposed to laser therapy without c-phycocyanin were considered as control.

### 3.7. Morphology of PDT Treated Cells

To observe the morphology of treated and control cells, the pre-seeded MBA-MD-231 cells in a 12-well plate were treated with different concentrations of c-phycocyanin and the cell-containing plate was exposed to LLLT laser treatment for 30 min. After laser treatment, the cells were incubated for 24 h and finally observed under a bright field microscope.

### 3.8. Acridine Orange-Ethidium Bromide Staining

To distinguish the cell death by PDT treatment, the pre-seeded MBA-MD-231 cells were treated with different concentrations of c-phycocyanin for 6 h, and then cells were irradiated under a 625-nm laser for 30 min with a 1 min intervals every 5 min. The treated cells were incubated for another 24 h and stained with acridine orange and ethidium bromide with a 1:1 ratio. After removing excess stain with PBS, cells were observed under a fluorescence microscope to differentiate the morphology of treated cells.

### 3.9. Hoechst Staining

The pre-seeded MBA-MD-231 cells (1 × 10^5^/well) in a 12-well plate were treated with c-phycocyanin and further irradiated with 625-nm laser for 30 min. After finishing 24 h incubation, cells were stained with Hoechst stain and rinsed with PBS buffer, and then the cells were observed under a fluorescence microscope.

### 3.10. DCFH-DA Staining

DCFH-DA is a commonly used cell-permeable stain to measure intracellular ROS. DCFH-DA converted to dichlorodihydrofluorescein (DCFH) by intracellular esterase. DCFH formed a fluorescent product of dichlorofluorescein (DCF) upon reaction ROS inside the cells. The pre-seeded MBA-MD-231 cells were treated with different concentrations of c-phycocyanin and irradiated under the LLLT system at 625-nm for 30 min. After 1 h of laser treatment cells were incubated with 20 μM of DCFH-DA for 30 min, then the cell-containing plate was read at an excitation/emission of 480/530-nm on a microplate reader. Then cells were observed under a fluorescence microscope for capturing images of the laser treated cells.

### 3.11. Rho-123 Staining

Rho-123 is a cationic lipophilic stain, which is readily sequestered by mitochondrial membranes and reflects mitochondrial healthiness. To analyze mitochondrial membrane potential, the MBA-MD-231 cells were seeded in a 12-well plate at 1 × 10^5^/well and treated with different concentrations of c-phycocyanin for 6 h. Then cells were treated with 625-nm laser for 30 min. After 24 h of laser treatment the medium in the culture plate were aspirated and cells were stained with 20 mM of Rho-123 for 15–20 min. The cells treated under laser without c-phycocyanin were maintained as a control. Then the culture plate was rinsed with PBS and cells were visualized under a fluorescence microscope.

### 3.12. Annexin V-FITC Staining

Phosphatidylserine translocation from the inner to outer leaflet of the plasma membrane is an early apoptotic feature. Cell surface phosphatidylserine can be detected by phosphatidylserine binding with an Annexin V-Cy3 apoptosis detection kit. Briefly, MBA-MD-231 cells (1 × 10^5^/well) were grown in a 12-well plate and treated with c-phycocyanin for 6 h. Then cells were exposed to 625-nm laser treatment for 30 min and incubated for 6 h in a CO_2_ incubator. After incubation cells were stained with double-staining solution (containing 1 μg·mL^−1^ Annexin V-Cy3 and 100 μM 6-carboxinflurescein diacetate (6-CFDA)) for 10 min at room temperature in the dark. Cells were then washed with 1× binding buffer followed and observed under a fluorescence microscope.

### 3.13. Statistical Analysis

All of the data is presented as mean ± standard deviation (SD). A comparison using (OriginPro 8, Northampton, MA, USA) (one-way analysis of variance) was performed to estimate the statistical parameters.

## 4. Conclusions

C-phycocyanin is a non-toxic, safe, and water-soluble brilliant blue pigment, which can produce enough ROS under laser exposure to kill cancer cells. The LLLT system also has the advantage of safety over other tissue-ablating laser systems. In addition, we found that the LLLT excited c-phycocyanin induced comparatively higher cell death to MDA-MB-231 breast cancer than HEK-293 cells. C-phycocyanin is a natural pigment and it is an extract of edible *Spirulina* algae. Unlike other commercial photosensitizers, c-phycocyanin does not cause toxicity to the cells in the absence of light and it has been used as a nutritional agent in the food industry. Further, tumor target-mediated c-phycocyanin-loaded vehicles need to be developed to achieve a tumor-specific laser therapy.

## Supplementary Materials

Supplementary materials can be accessed at: http://www.mdpi.com/1420-3049/21/11/1470/s1.
