# Supplementary material for: In Vitro Photodynamic Effect of Phycocyanin against Breast Cancer Cells
Source: Molecules. 2016 Nov 3;21(11):1470. doi: 10.3390/molecules21111470 (PMC6273603; doi:10.3390/molecules21111470)
Supplement: Supplementary file 1 [file molecules-21-01470-s001.pdf]

Supplementary Materials: In Vitro Photodynamic Effect of Phycocyanin against Breast Cancer Cells

Subramaniyan Bharathiraja, Hansu Seo, Panchanathan Manivasagan, Madhappan Santha Moorthy, Suhyun Park and Jungwan Oh

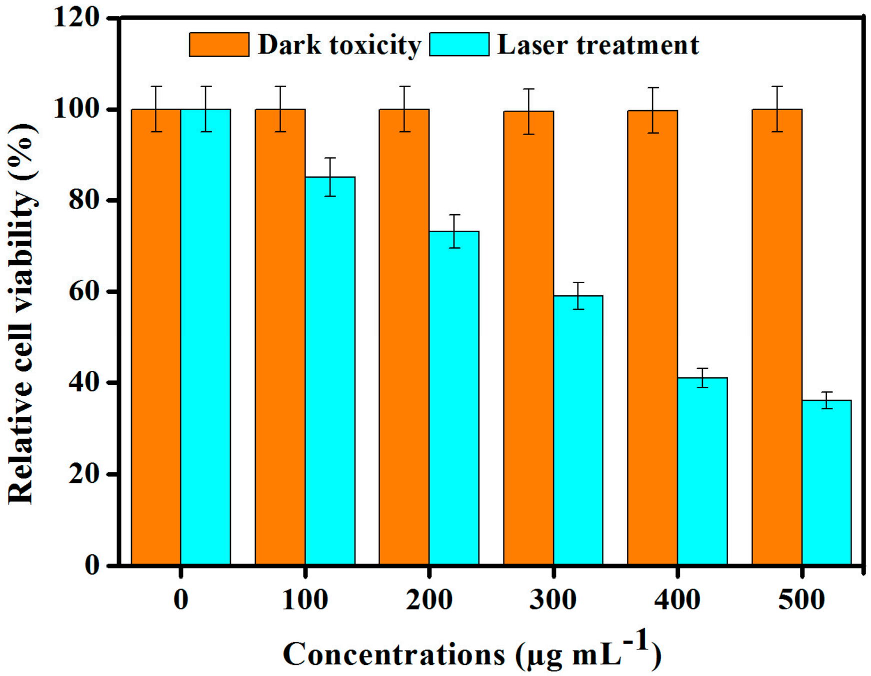

Figure S1. HEK-293 cell viability percentage was measured by MTT assay. Cell viability percentage is expressed as a value relative to that of the c-phycocyanin untreated cells which are set to 100%. Cell viability was not disturbed in the absence of light and it was reduced after PDT treatment using 625-nm laser treatment at 80 mW·cm^{-2} power density for 30 min.

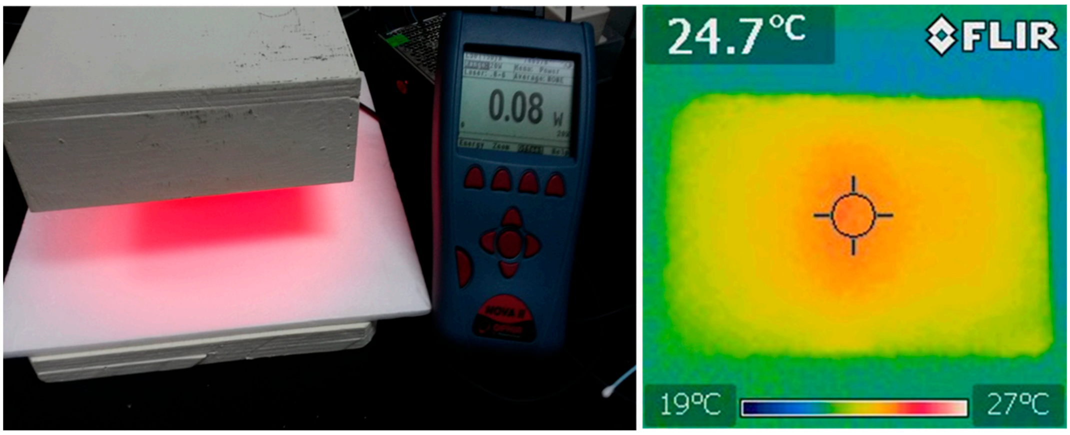

Figure S2. The 625-nm laser setup with 80 mW power density and infrared photograph showed the temperature level of 96-well plate with MBA-MD-231 cells after 30 min laser exposure.

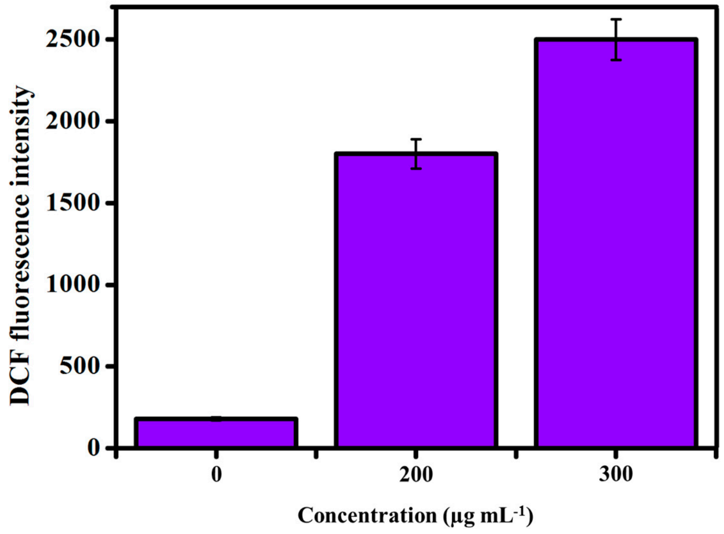

Figure S3. The increasing fluorescence intensity of DCF in different concentrations of c-phycocyanin sensitized MBA-MD-231 cells after PDT treatment using 625-nm laser at 80 mW·cm^{-2} for 30 min.

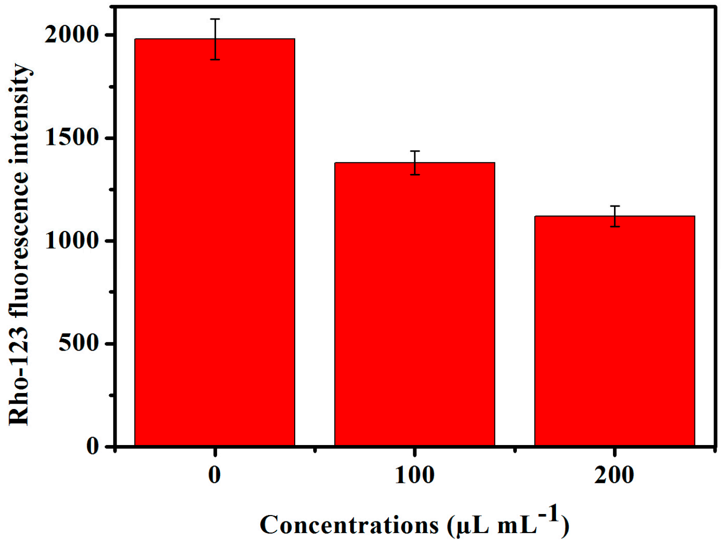

Figure S4. The decreasing fluorescence intensity of Rho-123 in different concentrations of c-phycocyanin sensitized MBA-MD-231 cells after PDT treatment using 625-nm laser at 80 mW·cm^{-2} for 30 min.
